# Oh lazy sister, where art thou?

**DOI:** 10.1038/s42003-021-02933-1

**Published:** 2021-12-13

**Authors:** Manuel Breuer

**Affiliations:** Communications Biology, https://www.nature.com/commsbio

## Abstract

Mitotic divisions achieve equal re-partition of chromosomes into daughter cells. In their recent work in *Developmental Cell*, Sen, Harrison et al. propose that the risk of mis-segregation in human mitotic cells is higher than previously thought and identify the existence of an early-anaphase correction mechanism. The study documents kinetochore dynamics in unprecedented detail, providing a detailed look at the events preceding loss of correct chromosomal numericity and genomic stability.

The fundamental importance of cell division lies in equal distribution of sister chromatids into budding daughter cells, ensuring genomic integrity. Failure to do so results in aneuploidies and the potential rise of micronuclei as distinct entities from daughter nuclei, both ultimately leading to genomic and transcriptomic instability and the promotion of cancer. During mitotic anaphase, chromatids that lag behind are the main culprits for mis-segregation, which are caused by improper attachments of kinetochores such as merotelic attachments (where one or both sister kinetochores are linked to opposing poles). Previously, the rate of lagging chromosomes in human cells was estimated at 5%; however, imaging limitations and data relying on fixed samples provide an incomplete picture. Using lattice light-sheet imaging and computational analysis of 3D kinetochore trajectory tracking, Andrew McAinsh, Nigel Borroughs and colleagues^1^ propose that the risk of mis-segregation in human mitotic cells is higher than previously assumed. They quantify metaphase sister chromatid behaviour as a predictor for lagging chromosomes in anaphase and collect evidence for the existence of an early anaphase correction mechanism orchestrated by the kinase Aurora B.

**Figure:**
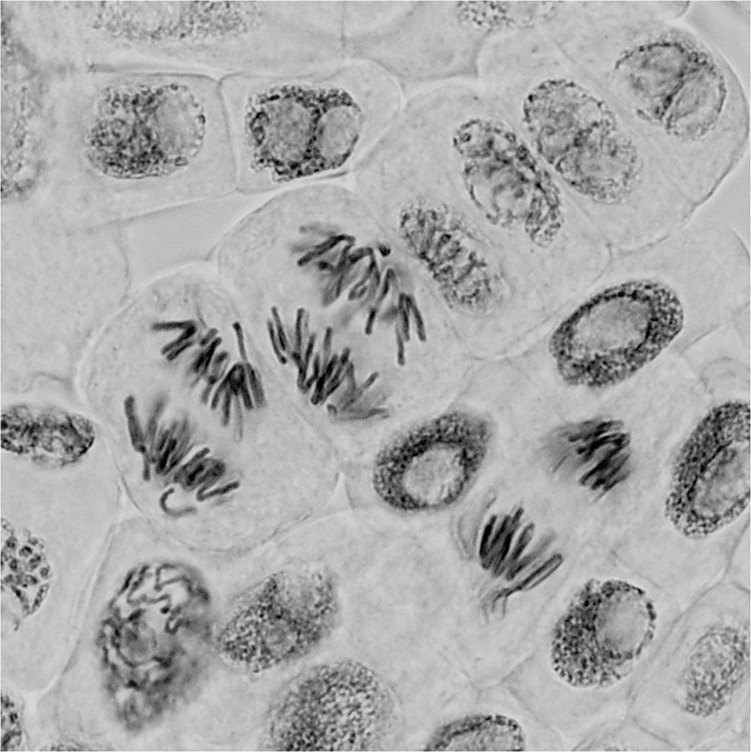
Getty Images, artist: alanphillips

The tracking pipeline developed by the authors allows for a quantifiable definition of lagging chromosomes, and they coin the term ‘laziness’ for the distance a kinetochore takes from its segregating cluster above a certain threshold. In doing so, Sen, Harrison et al. find that the number of cells with lazy, as opposed to timely, kinetochores and thus at risk of mis-segregation at anaphase, is at 18%—a value 2–3x higher than previously assumed. Connecting the behaviour of sister chromatids in metaphase to their lagging state in anaphase, they find that a small distance between sister chromatids is a strong predictor of lagging in anaphase; more importantly, kinetochores with reduced oscillatory dynamics are persistently lazy in anaphase. The authors then compare the shape of kinetochores to their behaviour and find stretched kinetochores, often associated with merotelic attachments, are more likely to be persistently lazy in anaphase.

To investigate an error correction mechanism of kinetochore-microtubule attachments, the authors then examine the role of the Aurora B kinase in anaphase. Upon the inhibition of Aurora B, they find an increase of lazy (and stretched) kinetochores. Further, kinetochores oriented towards the midzone display a preferential accumulation of Aurora B-dependent phosphorylation, a readout for error-correcting attachment destabilization. Interestingly, experimentally preventing Aurora B’s localization to the anaphase spindle midzone boosts the occurrence of micronuclei, a potentially catastrophic outcome to chromosome mis-segregation.

This work provides a comprehensive view of the dynamic behaviour of kinetochores and sister chromatids through mitotic meta- to anaphase, identifying a high occurrence of lagging chromosomes in dividing human cells. Aurora B, besides its well-known pre-anaphase error correction role, also monitors chromosome position in anaphase for accurate daughter cell formation. The authors here assign an early-anaphase correction role to Aurora B via an attachment-destabilising gradient. Thus, the manuscript establishes that nearly one fifth of dividing human cells have lazy chromosomes, and continuous monitoring by Aurora B at time of separation prevents the worst. However, as the authors point out, the impact of spindle forces in anaphase and the recent finding that Aurora B stabilizes kinetochore-microtubule attachments in anaphase will have to be reconciled with these findings in future work, as well as the direct demonstration of micronuclei formation and microtubule occupancy at kinetochores for lazy chromosomes.
